# Zinc Based Metal-Organic Frameworks as Ofloxacin Adsorbents in Polluted Waters: ZIF-8 vs. Zn_3_(BTC)_2_

**DOI:** 10.3390/ijerph18041433

**Published:** 2021-02-03

**Authors:** Doretta Capsoni, Giulia Guerra, Constantin Puscalau, Federica Maraschi, Giovanna Bruni, Francesco Monteforte, Antonella Profumo, Michela Sturini

**Affiliations:** 1 C.S.G.I. (Consorzio Interuniversitario per lo Sviluppo dei Sistemi a Grande Interfase) & Department of Chemistry, Physical Chemistry Section, University of Pavia, 27100 Pavia, Italy; doretta.capsoni@unipv.it (D.C.); constantin.puscalau@nottingham.ac.uk (C.P.); giovanna.bruni@unipv.it (G.B.); francesco.monteforte01@universitadipavia.it (F.M.); 2 Department of Chemistry, University of Pavia, 27100 Pavia, Italy; giulia.guerra01@universitadipavia.it (G.G.); federica.maraschi@unipv.it (F.M.); antonella.profumo@unipv.it (A.P.); 3 The GlaxoSmithKline Neutral Laboratories for Sustainable Chemistry, University of Nottingham, Jubilee Campus, Nottingham NG7 2TU, UK

**Keywords:** fluoroquinolone antibiotic, zinc-based metal-organic frameworks, adsorption, wastewater treatment, MOFs reusability, polluted waters

## Abstract

Two different zinc-based metal-organic frameworks (MOFs) were investigated to remove one of the most used fluoroquinolone antibiotic, Ofloxacin (OFL), from polluted water. The most common zeolitic imidazolate framework-8 (ZIF-8) and the green Zn(II) and benzene-1,3,5-tri-carboxylate (Zn_3_(BTC)_2_) were prepared through a facile synthetic route and characterized by means of Fourier-Transform Infrared (FT-IR) Spectroscopy, X-ray Powder Diffraction (XRPD), and Scanning Electron Microscopy (SEM) analyses. The two MOFs were compared in terms of both adsorption and kinetic aspects under real conditions (tap water, natural pH). Results showed that OFL was adsorbed in remarkable amounts, 95 ± 10 and 25.3 ± 0.8 mg g^−1^ on ZIF-8 and Zn_3_(BTC)_2_, respectively, following different mechanisms. Specifically, a Langmuir model well described the ZIF-8 profile, while for Zn_3_(BTC)_2_, cooperative adsorption occurred. Moreover the kinetic results were quite different, pseudo-second-order and sigmoidal, respectively. The suitability of ZIF-8 and Zn_3_(BTC)_2_ as adsorbent phases for water depollution was tested on tap water samples spiked with OFL 10 µg L^−1^. The obtained removal efficiencies, of 88% for ZIF-8 and 72% for Zn_3_(BTC)_2_, make these materials promising candidates for removing fluoroquinolone antibiotics (FQs) from polluted waters, notwithstanding their limited reusability in tap water, as demonstrated by in-depth characterization of the two MOFs after usage.

## 1. Introduction

The widespread diffusion of antibiotics in the environment, especially in the aquatic one, is a compelling issue. Indeed, they have been widely detected, mainly in their native form, in wastewater, surface water, drinking water, and soils in most, if not all, developed countries, especially those close to highly urbanized areas [1,2]. Detailed monitoring, carried out on influents and effluents from different urban wastewater treatment plants (WWTPs), located in urbanized and industrialized areas, on rivers that directly receive the discharge from plants, and on those located around the city, demonstrated that standard wastewater treatment plants are ineffective in removing such highly persistent emerging contaminants (EC) [1,2]. Consequently, antibiotic residues re-enter the hydrogeological cycle after the treatment at concentrations ranging from a few tens to hundreds of nanograms per liter [2,3,4,5]. These low concentrations are, however, high enough to cause well-known collateral effects, such as the generation of antibiotic-resistant bacteria (ARB) and antibiotic-resistant genes (ARG) [1,6]. It has been recently reported that reducing antibiotics consumption would not be enough to slow down the dissemination of resistance [6]. For this reason, the recent EU directive (2015/495/EU) has been updated (2018/840/EU), both to implement the current legislation and provide new proposals, such as the “action plan against the rising threats from antimicrobial resistance” specifically intended to regularly monitor antibiotics and, as well as, to reduce their widespread distribution [1].

Currently, there is an increasing commitment to finding efficient processes for the removal of antibiotic residues before they enter the aquatic compartment. Different treatment processes, both biological and physicochemical, have been proposed to improve conventional WWTPs [7]. The biological ones have been mainly used for antibiotic removal from wastewater and sludge. In contrast, the further ones, such as activated carbon adsorption, ozonization, advanced oxidation methods, nanofiltration, etc., have been tested for surface and drinking waters and wastewater effluents ([7] and references therein). A promising option is represented by the adsorption process that indeed is a good choice due to the many advantages it offers: (i) Low cost, (ii) sustainability, (iii) no generation of toxic byproducts, (iv) easy to integrate to conventional WWTPs, (v) easy to manage and maintain, (vi) possibility to exploit new adsorbent phases [7]. 

Over the last decade, research and development of new adsorbent materials have been expanded upon. A detailed examination of the literature has shown that different substrates, both natural and properly synthesized or modified, such as activated carbons, biochar from low-cost materials, functionalized nanotube, graphene oxide, mesoporous carbons, and clay minerals were used for antibiotic adsorption [7,8,9,10]. Interestingly, the recent employment of metal-organic frameworks (MOFs) as adsorbent phases makes them an alternative and a promising option for remediation of polluted waters [11,12,13]. MOFs consist of metal transition ions orderly linked to organic ligands. Besides their intrinsic properties (high surface area and porous structure), they have different functionalities, making them versatile materials. In this way, they can adequately interact with different molecules through electrostatic interactions, H-bonding, hydrophobic interaction, and π- π stacking. Several MOFs have been recently employed for drug remediation, such as H-KUST 1 (H-KUST: Hong Kong University of Science and Technology), MIL-53, MIL-100, MIL-101 (MIL: Materiaux de l’Institut Lavoisier), UiO-66 (UiO: Universitetet i Oslo), and ZIFs (ZIF: Zeolitic Imidazolate Framework), showing remarkable affinities towards those organic molecules [14,15,16,17,18,19,20,21,22,23]. However, the effect of some key parameters, such as the use of water not previously purified, natural pH, and µg per liter drug concentration, seems not to have been deeply investigated yet.

Thus, we deemed it appropriate to study and compare the efficiency of two different zinc-based metal-organic frameworks, the largely used zeolitic imidazolate framework-8 (ZIF-8) and the Zn(II) and benzene-1,3,5-tri-carboxylate (Zn_3_(BTC)_2_), for the removal of a widely used human antibiotic, Ofloxacin (OFL), under relevant real conditions, viz. tap water and low-level concentration. OFL was selected as a model molecule due to our previous investigation on fluoroquinolones (FQs) affinity towards natural and synthetic substrates [8,9,10]; ZIF-8 and Zn_3_(BTC)_2_ are suitable for water remediation due to a non-toxic metal ion, Zn^2+^. ZIF-8 displays a sodalite-type crystal structure and large pores with diameters of 11.6 Å accessible through small apertures with a diameter of 3.4 Å [24]. It is water-stable due to a strong coordination bond, according to the hard/soft acid/base theory [25]. Moreover, compared to other MOFs, it shows exceptional thermal and chemical stability [26] in aqueous and harsh environments. The high surface area and pore size make it attractive in lots of applications such as gas storage, separations, catalysis, drug delivery and chemical sensors [27]. Zn_3_(BTC)_2_ was chosen for its high water solution stability, biocompatibility, and green synthesis (sonochemical process). Moreover, while already applied in molecular sensing [28] and drug delivery [29], to our knowledge, it was not yet used for water remediation. It displays a monoclinic crystal structure with elliptically-shaped pores of 4 × 5 Å diameter [30]. To our knowledge, only in the case of ZIF-8, a recent study addressed the removal of OFL under controlled laboratory conditions [20], quite different from the ones mentioned above. In this study, the two MOFs were compared in terms of both adsorption and kinetic aspects under real conditions, micrograms per liter concentration, and tap water, not yet investigated, and, importantly, not yet fully characterized after use.

## 2. Materials and Methods

### 2.1. Materials

All the chemicals employed were reagent grade or higher in quality. OFL, ultrapure hydrochloric acid (HCl) 30%, Zn(NO_3_)_2_·6H_2_O, 2-methylimidazole, Zn(CH_3_COO)_2_·2H_2_O, and trimesic acid (H_3_BTC) were purchased from Merck (Milano, Italy). High Performance Liquid Chromatography (HPLC) gradient–grade acetonitrile (ACN) and absolute ethanol were purchased by VWR International (Milano, Italy), H_3_PO_4_ (85%w/w), methanol for Liquid Chromatography Mass Spectrometry LC/MS, and water for LC/MS by Carlo Erba Reagents (Cornaredo, Milano, Italy). Aqueous OFL solution (305 mg L^−1^) was prepared in tap water and stored in the dark at 4 °C before use.

### 2.2. Synthesis

#### 2.2.1. ZIF-8

ZIF-8 was synthesized by following the procedure reported by Pan et al. [31], the first example of ZIFs synthesis in a purely aqueous system. This synthetic approach was chosen becouse it is rapid and performed at room temperature. Compared to other synthesis routes reported in the literature to prepare ZIF-8 [27], it is not time-consuming and does not require organic solvents and high energy-consuming steps. A solution with Zn^2+^:2-methylimidazole:H_2_O in 1:70:1238 molar ratio was prepared as follows. Firstly, 0.293 g Zn(NO_3_)_2_·6H_2_O was dissolved in 2 g distilled water (DI). Secondly, 5.675 g 2-methylimidazole was dissolved in 20 g DI water. The obtained solutions were then mixed and stirred at room temperature. The synthesis solution turned milky almost instantly after the two solutions were mixed. After stirring for 5 min, the product was collected by centrifugation and washed with DI water several times. The obtained powder was dried at 70 °C overnight. The activation process was then carried out to remove the solvent molecules from the ZIF-8 pores: The powder was heated under vacuum at 80 °C for 3 h and stored at room temperature in an Ar-filled dry-box (MBraun, Garching, Germany; O_2_ ≤ 1ppm, H_2_O ≤ 1 ppm). 

#### 2.2.2. Zn_3_(BTC)_2_

The Zn_3_(BTC)_2_ was prepared following the sonochemical synthesis reported by Lestari et al. [29]. 3.133 g Zn(CH_3_COO)_2_·2H_2_O was dissolved in 25 mL DI water, and 2 g trimesic acid was dissolved in 25 mL Ethanol (EtOH). The obtained solutions were mixed and sonicated for 60 min. The obtained white precipitate was washed several times with EtOH and dried at room temperature overnight. No activation process was carried out due to the loss of crystallinity by treating the powder under vacuum at 80 °C, as demonstrated by X-ray powder diffraction technique. 

### 2.3. Characterization Techniques

X-ray powder diffraction (XRPD) measurements were performed by using a Bruker D5005 diffractometer with the CuKα radiation (Bruker, Karlsruhe, Germany), graphite monochromator, and scintillation detector. The patterns were collected in the 5–25° and 5–35° angular range for the ZIF-8 and Zn_3_(BTC)_2_ samples, respectively, step size of 0.03° and counting time of 4s/step. A silicon low-background sample holder was used. An air-tight dome sample holder was used for the activated ZIF-8.

Fourier-Transform Infrared (FT-IR) spectra were obtained with a Nicolet FT-IR iS10 Spectrometer (Nicolet, Madison, WI, USA) equipped with an ATR (attenuated total reflectance) sampling accessory (Smart iTR with ZnSe plate) by co-adding 32 scans in the 4000–500 cm^−1^ range at 4 cm^−1^ resolution. 

Scanning Electron Microscopy (SEM) measurements were performed using a Zeiss EVO MA10 (Carl Zeiss, Oberkochen, Germany) Microscope. The SEM images were collected on gold-sputtered samples.

### 2.4. Thermodynamic and Kinetic Studies

#### 2.4.1. Thermodynamic Experiments

OFL adsorption on the two MOFs was studied using a batch equilibration method. Antibiotic solutions in the range 8–305 mg L^−1^ were prepared in tap water. Ten mg of each MOF was weighed into separated flasks and mixed with 20 mL of OFL solution. In the case of ZIF-8, the antibiotic solution was added using a syringe into the sealed flasks containing the activated adsorbent phase removed from the dry box before use, and the solution pH was adjusted around 8, adding 50 µL 1 M HCl. The flasks, wrapped with aluminum foils to prevent FQ light-induced decomposition, were shaken at 90 rpm for 24 h at room temperature to ensure the equilibration. The suspensions were filtered through a 0.22 µm nylon syringe filter and analyzed by HPLC–UV Shimadzu (Shimadzu Corporation, Milano, Italy) to determine OFL concentration at equilibrium (*C_e_*). 

The following Equation (1) calculated the adsorbed OFL amount at equilibrium (*q_e_*, mg g^−1^):

(1) $$q_{e}=\frac{\left(C_{0}-C_{e}\right)\times V}{M}$$

where *C*_0_ is the initial OFL concentration (mg L^−1^), *C_e_* the OFL concentration in solution at equilibrium (mg L^−1^), *V* the volume of the solution (L), and *M* the amount of the adsorbent (g).

All experiments were performed in triplicate with good reproducibility (RSD ≤ 10%). 

No change in OFL concentration was detected in the control samples (tap water solutions containing OFL and no adsorbent phase). 

The isotherm parameters were calculated by dedicated software (OriginPro, Version 2019b. OriginLab Corporation, Northampton, MA, USA).

#### 2.4.2. Kinetic Experiments

Twenty mg of ZIF-8 and 20 mg of Zn_3_(BTC)_2_ were suspended in 40 mL of tap water at an initial OFL concentration of 36 mg L^−1^ and 12 mg L^−1^, respectively. OFL initial concentrations were chosen according to the thermodynamic results to guarantee an adsorption efficiency in the range of 10–85%, as indicated in the guidelines ASTM D3860 [32]. As mentioned above, in the case of ZIF-8, the antibiotic solution was added using a syringe into the sealed flasks containing the activated adsorbent phase removed from the dry box before use. Then it was acidified, adding 50 µL 1 M HCl to reach a pH value around 8, while no pH modification was required for Zn_3_(BTC)_2_ suspension. The suspensions were maintained under magnetic stirring (700 rpm) throughout the experiment. One hundred µL supernatants were collected at planned times in the range 0–60 min, diluted to 5–10 mL tap water, filtered (0.22 µm nylon syringe filter), and injected in the HPLC-FD (Perkin Elmer, Milano, Italy) system to determine the OFL concentration at time *t* (*C_t_*).

The following Equation (2) calculated the adsorbed OFL amount at time *t* (*q_t_*, mg g^−1^):

(2) $$q_{t}=\frac{\left(C_{0}-C_{t}\right)\times V}{M}$$

where *C*_0_ is the initial OFL concentration (mg L^−1^), *C_t_* the OFL concentration in solution at time *t* (mg L^−1^), *V* the volume of the solution (L), and *M* the amount of the adsorbent (g).

All experiments were performed in triplicate with good reproducibility (RSD ≤ 10%). No change in OFL concentration was detected in the control sample (tap water solutions containing OFL and no adsorbent phase). 

The kinetic parameters were calculated by dedicated software (OriginPro, Version 2019b. OriginLab Corporation, Northampton, MA, USA).

### 2.5. Analytical Measurements

For the adsorption experiments, an HPLC-UV Shimadzu (Shimadzu Corporation, Milano, Italy) system consisting of an LC-20AT solvent delivery module equipped with a DGU-20A3 degasser and interfaced with an SPD-20A UV detector, was used. The wavelength selected for analysis was 280 nm corresponding to the maximum OFL adsorption. Each sample was filtered (0.22 µm) and injected (20 µL) into a 250 × 4.6 mm, 5 µm KromaPhase 100 C18 (Scharlab, Riozzo di Cerro al Lambro, Milano, Italy) coupled with a similar guard-column. Isocratic elution was carried out by using a mobile phase 25 mM H_3_PO_4_–ACN (85:15). The flow rate was 1.0 mL min^−1^.

Calibration with four standards at concentrations between 1 and 10 mg L^−1^ yielded optimal linearity (R^2^ > 0.9996). The quantification limit was 0.8 mg L^−1^.

The HPLC system used for the kinetic experiments and OFL adsorption at 10 µg L^−1^ consists of a pump Series 200 (Perkin Elmer, Milano, Italy) equipped with a vacuum degasser and a programmable fluorescence detector (FD). The fluorescence excitation/emission wavelengths selected were 280/500 nm. Fifty µL of each sample were filtered (0.22 µm) and injected into a 250 × 4.6 mm, 5 µm Ascentis RPAmide (Supelco-Merck Life Science, Milano, Italy) coupled with a similar guard-column. The mobile phase was 25 mM H_3_PO_4_–ACN (85:15), a flow rate of 1 mL min^−1^.

Calibration with four standards at concentrations between 1 and 10 µg L^−1^ yielded optimal linearity (R^2^ > 0.9996). The quantification limit was 0.9 µg L^−1^.

## 3. Results and Discussion

In the present work, two different zinc-based MOFs, ZIF-8 and Zn_3_(BTC)_2_, were critically compared to remove OFL antibiotic under relevant real conditions and characterized before and after their use. 

### 3.1. MOFs Characterization

#### 3.1.1. ZIF-8

**Figure 1:**
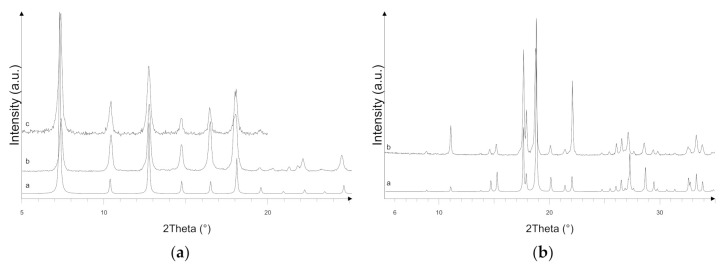
X-ray powder diffraction (XRPD) of (**a**) zeolitic imidazolate framework-8 (ZIF-8) and (**b**) Zn(II) and benzene-1,3,5-tri-carboxylate (Zn_3_(BTC)_2_): simulated (pattern a), as prepared (pattern b), and activated (pattern c). a.u. = arbitrary units

In Figure 1a, the X-ray powder diffraction pattern of the ZIF-8 sample is shown, compared to the one simulated on the basis of the crystal structure reported in the literature [33] and deposited in the Cambridge Crystallographic Data Centre (CCDC 864310). The diffraction patterns display comparable peak positions and intensities, suggesting that the crystalline ZIF-8 sample has been successfully synthesized. The crystalline structure is preserved after activation, as confirmed by the diffraction pattern shown in Figure 1a (pattern c). 

**Figure 2:**
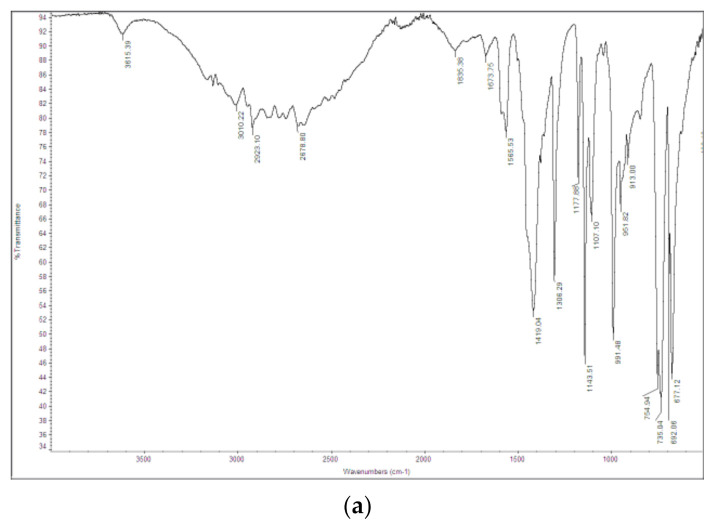

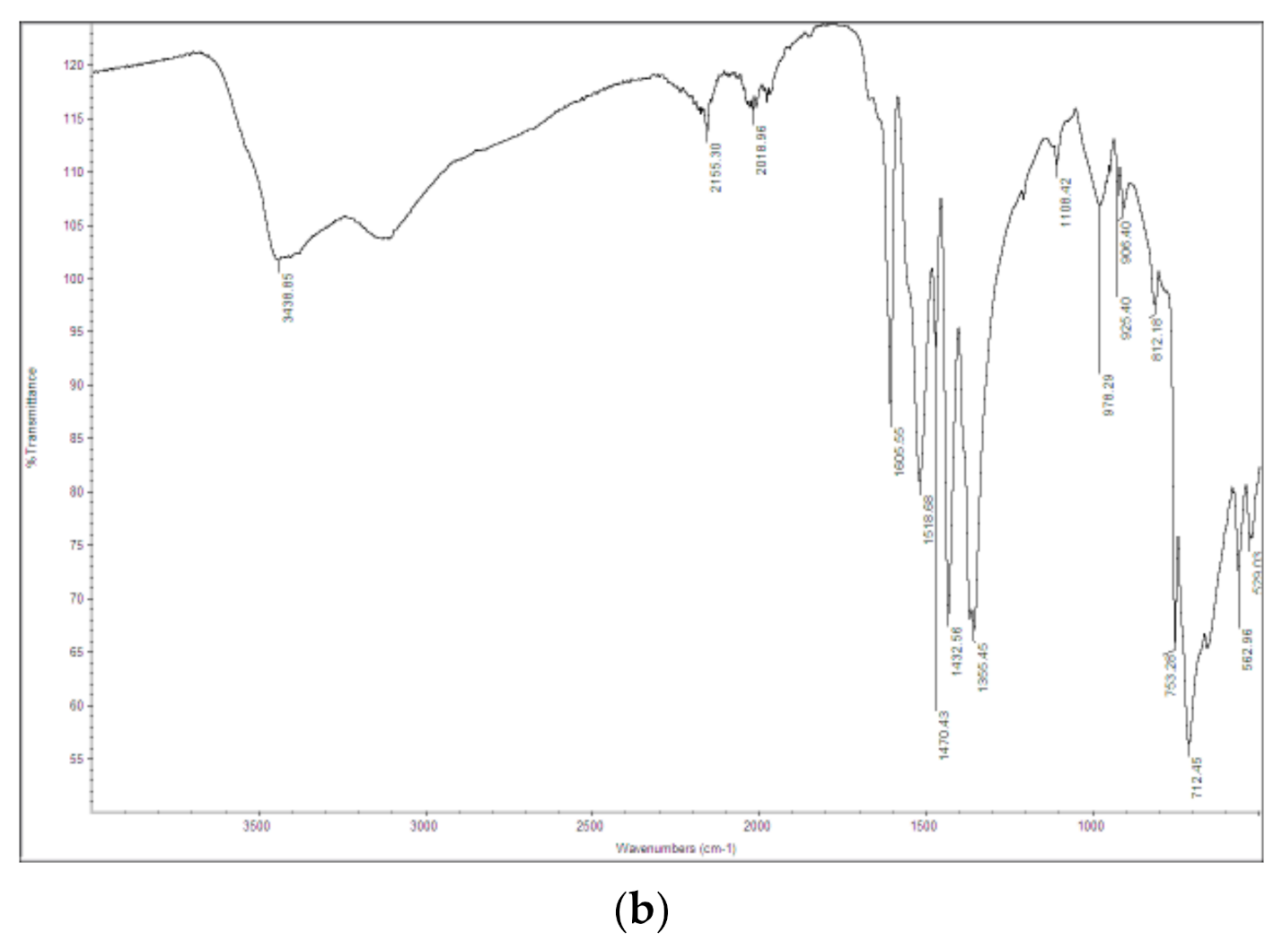
Fourier-Transform Infrared (FT-IR) spectra of (**a**) ZIF-8 and (**b**) Zn_3_(BTC)_2_ samples.

**Figure 3:**
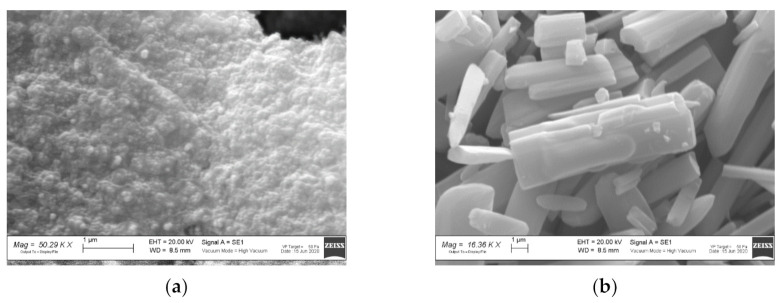
Scanning Electron Microscopy (SEM) images of (**a**) ZIF-8 and (**b**) Zn_3_(BTC)_2_ samples.

The FT-IR spectrum of the ZIF-8 sample is shown in Figure 2a. The observed bands are consistent with those reported in the literature for ZIF-8 [34,35,36,37]. The signals at 1673 and 1565 cm^−1^ are attributed to the C=C and C=N stretch modes, respectively. The bands at 1300–1460 cm^−1^ are due to the ring stretching. The band at 1143 cm^−1^ and the peaks at 991 and 754 cm^−1^ are assigned to the stretching mode of the aromatic C–N and to the C–N bending vibration and C–H bending modes, respectively. The band at 697 cm^−1^ is due to the ring out of plane bending vibration of the 2-methylimidazole. Figure 3a shows the SEM image of the ZIF-8 sample. Rounded particles with a homogeneous size of about 100 nm are observed. They are interconnected to form large agglomerates. 

#### 3.1.2. Zn_3_(BTC)_2_

The XRPD pattern of the Zn_3_(BTC)_2_ sample is shown in Figure 1b and compared to the simulated pattern obtained by the crystal structure reported in the literature [30] and deposited in the Cambridge Crystallographic Data Centre (CCDC-1274034). The diffraction patterns display comparable peak positions, suggesting that the crystalline Zn_3_(BTC)_2_ sample has been successfully prepared.

The FT-IR spectrum of the Zn_3_(BTC)_2_ sample is shown in Figure 2b. The signals are consistent with those reported in the literature for Zn_3_(BTC)_2_ [29]. The broad peaks observed at 3300–2700 cm^−1^ and 3450 cm^−1^ are attributed to the stretching vibration of O-H bonds of trimesic acid, indicating coordinated water molecules in the MOF. At 3438 cm^−1,^ the C-H aromatic bands are shown. The peak at 1518 cm^−1^ is attributed to the C=C bond in the aromatic ring. More interestingly, the -COOH absorption bands typically observed at 1730–1690 cm^−1^ are not detected, thus demonstrating that the trimesic acid is bound to the metal center. The band detected at 753 cm^−1^ is assigned to the Zn-O stretching vibration.

Figure 3b shows the SEM image of the Zn_3_(BTC)_2_ sample. It displays stick-like shaped particles with a smooth surface and irregular particle size: 2–10 µm in length, and 1–3 µm thickness.

### 3.2. Preliminary Experiments

Since both pH and ionic strength affect the adsorption process [17,20,24], a set of preliminary experiments were carried out to evaluate the feasibility of the adsorption process under real conditions. For this purpose, tap water was chosen as a solvent because of its invariant composition and a more remarkable similarity to environmental waters than the ultrapure one. In Appendix A, the physicochemical parameters of the municipal water collected in Pavia are shown. 

First, working pH was evaluated. 10 mg of ZIF-8 and Zn_3_(BTC)_2_ were separately suspended in 20 mL of tap water not containing the drug and shaken (90 rpm) for 24 h at room temperature. Then the suspensions were centrifuged, and the supernatants separated for the pH measurement. The measured pH values were 9.0 and 6.7, respectively. 

No additional treatment was necessary for Zn_3_(BTC)_2_ suspension, while small volumes of 1 M HCl were added to ZIF-8 suspension to adjust the pH in the range of 6.5–8.3, typical of environmental waters. Specifically, 8.1 and 6.7 pH values were obtained, adding 50 and 100 µL of 1 M HCl, respectively.

Previous investigations regarding ZIF-8 thermal and chemical stabilities showed its exceptional hydrolysis resistance in benzene solution, methanol, water, and aqueous sodium hydroxide [26]. On the contrary, its application was limited by dissolution under acidic conditions [38,39]. For this reason, we decided to add the smallest 1 M HCl volume tested and work at pH around 8. 

A further test was carried out in parallel on activated and not activated ZIF-8 following the procedure reported in Section 2.4.1. Each material (10 mg) was separately suspended in 9 mg L^−1^ OFL tap water solution. After 24 h equilibration, the drug percentage uptake resulted in 76 and 38%, respectively. Consequently, all experiments were carried out on the activated form.

On the contrary, Zn_3_(BTC)_2_ was used without activation to avoid any loss of crystallinity, as demonstrated by XRPD data collected on a sample activated at 80 °C (data not shown). 

Based on the recent literature regarding drug adsorption on MOFs [16,17,19,22], 10 mg of MOF was chosen for all the experiments. Yu and Wu [20] observed a decreased OFL adsorption capacity increasing ZIF-8 amount from 10 to 50 mg. 

### 3.3. Isotherm and Kinetic Studies

#### 3.3.1. Isotherm Studies

Adsorption isotherms are widely used to characterize the retention of chemicals in a solid phase, describing the maximum uptake and the relationship between the amount of the adsorbed molecule (*q_e_*) and the dissolved molecule’s concentration (*C_e_*) in the solution at equilibrium quantitatively. 

In this paper, the most frequently used models, i.e., Freundlich and Langmuir, and the sigmoidal and Brunauer-Emmett-Teller (BET) models, were applied to better describe OFL adsorption onto ZIF-8 and Zn_3_(BTC)_2_.

The Freundlich model, usually applied to non-ideal adsorption on the heterogeneous surface, is expressed by the following Equation (3):

(3) $$q_{e}=K_{F}C_{e}^{1/n}$$

where *K_F_* is the empirical constant indicative of adsorption capacity, and *n* is the empirical parameter representing the heterogeneity of site energies.

The Langmuir model (Equation (4)) assumes that the adsorption process occurs in a monolayer that covers the surface of the material:

(4) $$q_{e}=\frac{q_{m}K_{L}C_{e}}{1+K_{L}C_{e}}$$

where *K_L_* is the Langmuir constant and *q_m_* the monolayer saturation capacity.

The sigmoidal model (Equation (5)) usually describes cooperative adsorption, and it is expressed by the equation:

(5) $$q_{e}=\frac{q_{m}}{1+e^{-A\left(C_{e}-F\right)}}$$

where *q_m_* is the maximum amount of molecules adsorbed, *A* is a coefficient indicating the adsorption mechanism’s efficiency, and *F* represents the inflection point.

BET isotherm for liquid-phase adsorption (Equation (6)) was applied [40] to evaluate the adsorption equilibrium constant of potential upper layers (*K_L_*) along with the equilibrium constant for the first layer (*K_S_*):

(6) $$q_{e}=\;q_{m}\frac{K_{s}C_{eq}}{\left(1-K_{L}\right)\left(1-K_{L}C_{eq}+K_{s}C_{eq}\right)}$$

**Figure 4:**
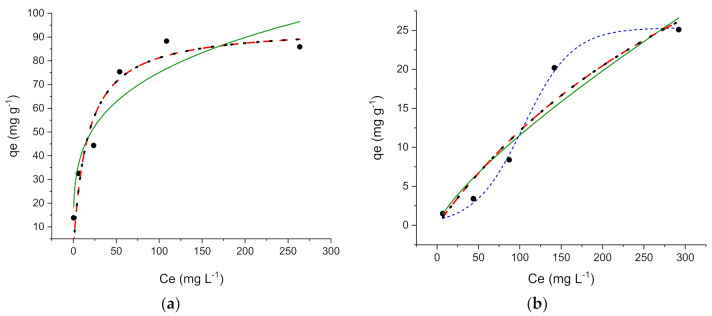
Adsorption profiles Langmuir (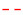), Freundlich (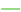), Brunauer-Emmett-Teller BET (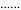), and sigmoidal (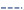) for Ofloxacin (OFL) on (**a**) ZIF-8 and (**b**) Zn_3_(BTC)_2_. (Experimental conditions: MOF (metal-organic framework) 10 mg, 20 mL OFL solution from 8 to 305 mg L^−1^).

As shown in Figure 4a,b, the experimental adsorption profiles of OFL on ZIF-8 and Zn_3_(BTC)_2_ are quite different, as well as their maximum uptake, 95 ± 10 mg g^−1^ for ZIF-8 and 25.3 ± 0.8 mg g^−1^ for Zn_3_(BTC)_2_. 

OFL adsorption took place on ZIF-8 clearly at specific homogeneous sites within the adsorbent, while a sigmoidal profile was observed for Zn_3_(BTC)_2_. 

The isotherm parameters were calculated by dedicated software and are listed in Table 1.

For ZIF-8, the correlation coefficient (R^2^) and the lower standard deviations of Langmuir parameters confirm that the drug adsorption occurred through a monolayer coverage. *K_S_* (0.06 ± 0.03) and *q_m_* values obtained by applying the BET model confirmed the monolayer adsorption process, and the *K_L_* value indicated that no multi-layer adsorption occurred.

The profile observed for Zn_3_(BTC)_2_ describes cooperative adsorption and indicates that at lower OFL concentration in the solution, the adsorption is low, increasing when the solute concentration increases. A similar trend was found in previous studies regarding the adsorption of organic molecules onto various adsorbent materials [8,9,10,41].

The experimental OFL equilibrium uptake (*q_m exp_*) is in good agreement with the calculated one for both the investigated materials, as shown in Table 1. Both ZIF-8 and Zn_3_(BTC)_2_ have a good affinity towards OFL and are competitive in term of adsorption capacity compared with other MOFs used for the same purpose. For example, the adsorption capacities ranges from 50 mg g^−1^ for UiO-66-NH_2_@RAMIP@BSA to 133 mg g^−1^ for UiO-67/CdS/rGO-x [42,43].

#### 3.3.2. Kinetic Studies

Various adsorption kinetic mechanistic models were used to describe OFL uptake on the two investigated MOFs, i.e., the most common pseudo-first-order (Equation (7)) and the pseudo-second-order (Equation (8)) equations, and a sigmoidal one (Equation (9)).

(7) $$q_{t}=q_{e}\left(1\;-\;e^{-k_{1}t}\right)$$

(8) $$q_{t}=\;\frac{\left(q_{e}^{2}k_{2}t\right)}{\left(1+q_{e}k_{2}t\right)}$$

(9) $$q_{t}=\frac{q_{e}}{1+e^{-A\left(C_{t}-F\right)}}$$

where *q_t_* and *q_e_* were the OFL amount adsorbed at time *t* and at equilibrium, respectively, *k*_1_ was the pseudo-first order rate constant, *k*_2_ the pseudo-second-order rate constant, *A* the sigmoidal efficiency adsorption constant, *F* the sigmoidal flex.

**Figure 5:**
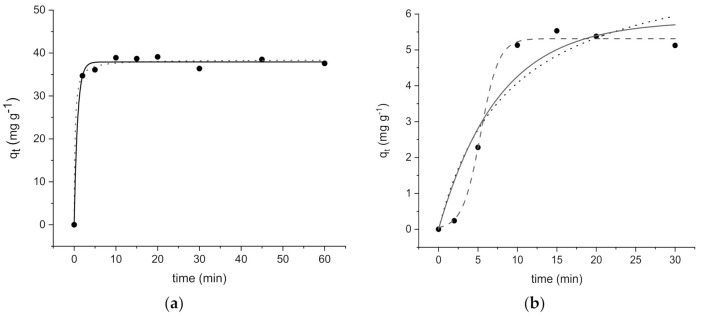
Kinetic profiles (pseudo-first-order (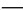), pseudo-second order (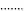), and sigmoidal (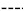)) for Ofloxacin (OFL) on (**a**) ZIF-8 (Experimental conditions: metal-organic frameworks (MOF) 20 mg, 40 mL tap water, OFL initial concentration 36 mg L^−1^) and (**b**) Zn_3_(BTC)_2_ (Experimental conditions: MOF 20 mg, 40 mL tap water, OFL initial concentration 12 mg L^−1^).

The experimental kinetic profiles are shown in Figure 5a,b and revealed a different trend under the same experimental conditions. 

The kinetic parameters obtained by fitting the experimental data are shown in Table 2.

Being the correlation coefficient (R^2^) for ZIF-8 always higher than 0.99, both Equations (7) and (8) models should describe the kinetics of the process. However, the pseudo-second-order model seems to fit the experimental data better than the pseudo-first-order, as shown in Figure 5a and reported by other authors [20].

On the contrary, in the case of Zn_3_(BTC)_2,_ the same kinetic models did not depict the experimental data, while the sigmoidal equation (Equation (9)) well fits the experimental data. A good R^2^ was obtained. 

According to these assessments, we can conclude that a fast uptake, lower than 10 min, occurs on both the studied MOFs.

### 3.4. Reusability and Post-Use Characterization of MOFs

#### 3.4.1. Reusability

After the first adsorption, both MOFs were separated and suspended for a second time in 20 mL tap water samples containing OFL 10 mg L^−1^, following the same procedure described for the adsorption isotherms (see Section 2.4.1). After 24 h equilibration, a negligible amount of antibiotic (<LOQ, Limit of Quantification) was adsorbed in both cases. Similar results were obtained after heating MOFs at 60 °C after the first adsorption cycle and before the second one.

To evaluate the reasons limiting the MOFs’ re-use, as demonstrated by the adsorption tests after the first use, both ZIF-8 and Zn_3_(BTC)_2_ have been characterized after OFL adsorption. 

#### 3.4.2. ZIF-8/Ofloxacin System

**Figure 6:**
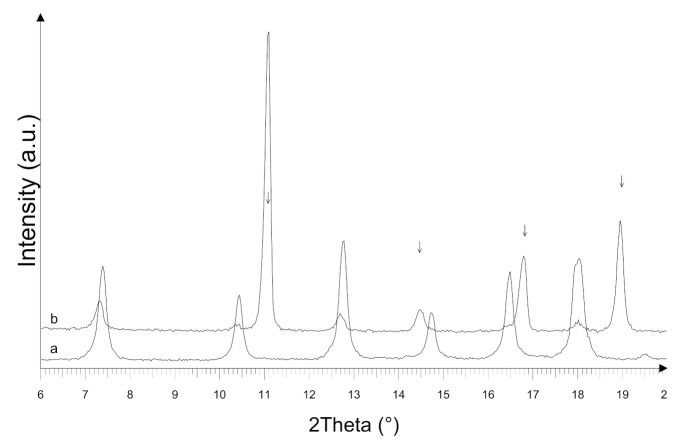
XRPD patterns of ZIF-8: As prepared (pattern a), after OFL adsorption at pH 8.1 (pattern b). The new peaks are indicated by arrows.

The ZIF-8 powder after the first OFL adsorption at pH 8.1 was recovered by centrifugation and dried. The diffraction pattern is shown in Figure 6 and compared to the as-synthesized one. In the sample after the antibiotic adsorption, new peaks are detected. The obtained results suggest that ZIF-8 underwent a degradation. The decomposition process is not complete, as weak peaks pertinent to the ZIF-8 are again observed in the pattern b.

**Figure 7:**
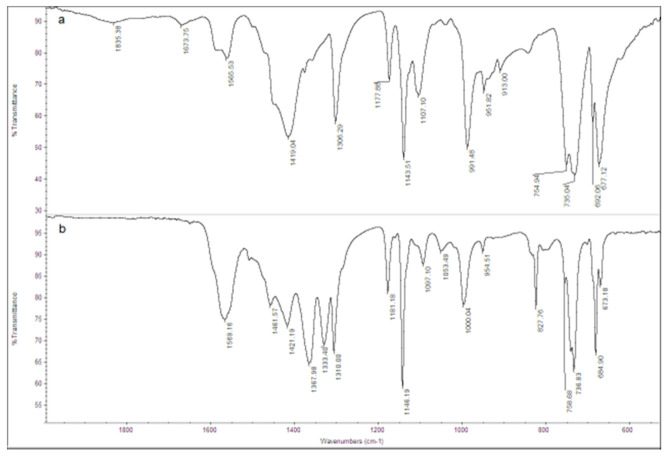
FT-IR spectra of ZIF-8: As-synthesized (spectrum a) and after OFL adsorption at pH 8.1 (spectrum b).

The observation is also supported by FT-IR and SEM results. The FT-IR spectrum of the post-use ZIF-8 is shown in Figure 7; some additional bands in the 1400–1300 cm^−1^ region are observed, suggesting a degradation process occurred in the ZIF-8/OFL system. The spectrum of the as-synthesized ZIF-8 is also reported.

**Figure 8:**
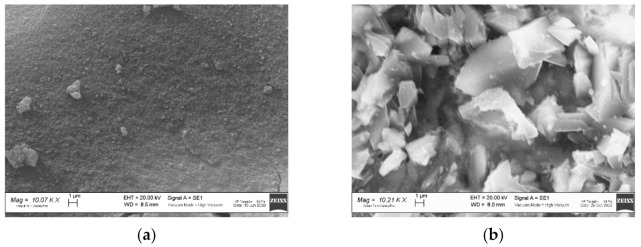
SEM images of the ZIF-8 samples: (**a**) As-synthesized and (**b**) after OFL adsorption at pH 8.1.

The SEM images of the ZIF-8 sample as-synthesized and after OFL adsorption at pH 8.1 are shown in Figure 8. Significant changes in particle morphology are detected after MOF is used. In particular, micrometric grains displaying flat surfaces are observed. The changes in morphology do not prove, but may be a consequence of, the occurrence of decomposition processes.

The degradation of the ZIF-8 structure after OFL adsorption at pH 8.1 limits its possible re-use. This agrees with the literature reporting the ZIF-8 instability by decreasing the pH [26,38,39] and with our results: We demonstrated by XRPD technique that a ZIF-8 soaked in an acidic environment (pH 6.7) completely decomposed (data not shown).

#### 3.4.3. Zn_3_(BTC)_2_/Ofloxacin System

**Figure 9:**
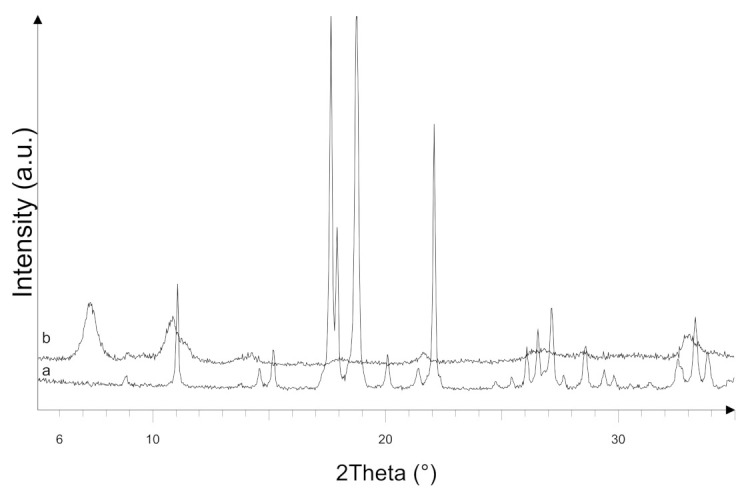
XRPD patterns of Zn_3_(BTC)_2_: As-synthesized (pattern a) and after OFL adsorption in tap water (pattern b).

Figure 9 shows the Zn_3_(BTC)_2_ diffraction pattern after OFL adsorption in tap water. The as-synthesized Zn_3_(BTC)_2_ pattern is also shown for comparison. The Zn_3_(BTC)_2_ diffraction peaks are not observed in the sample after use, while new broad peaks are detected, indicating the sample underwent amorphization and degradation.

**Figure 10:**
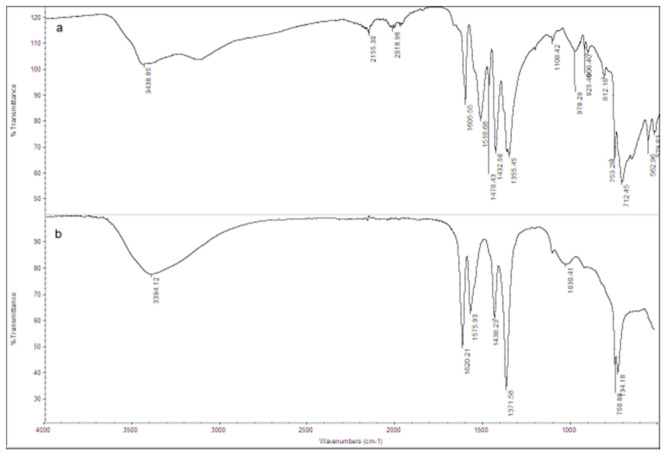
FT-IR spectra of Zn_3_(BTC)_2_: As prepared (spectrum a) and after OFL adsorption in tap water (spectrum b).

Figure 10 shows the FT-IR spectra of Zn_3_(BTC)_2_ as-synthesized and after OFL adsorption. Some changes are observed in the 1700-1300 cm^−1^ spectral region after use, confirming the degradation process occurred.

**Figure 11:**
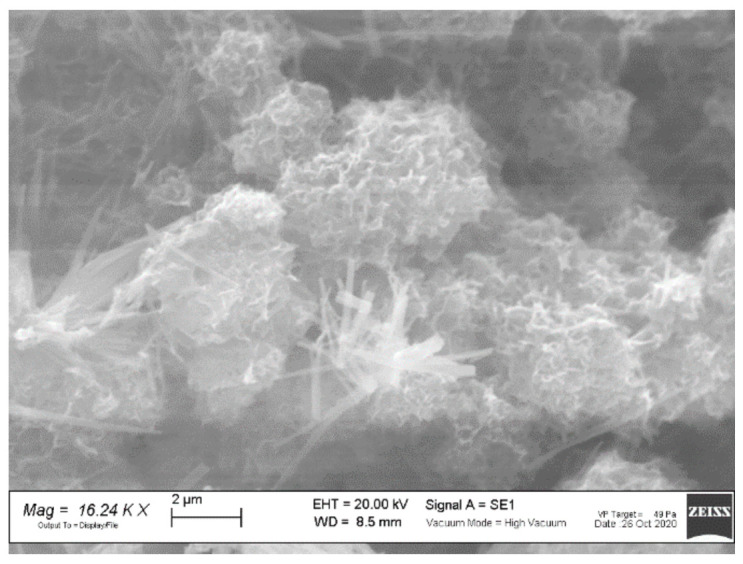
SEM image of the Zn_3_(BTC)_2_ sample after OFL adsorption in tap water.

The SEM image of the Zn_3_(BTC)_2_ after OFL adsorption in tap water is shown in Figure 11. An impressive change in particle morphology and grain size is detected after Zn_3_(BTC)_2_ use (see Figure 3b, as-prepared sample); this evidence supports the loss of crystallinity and MOF degradation evidenced by XRPD and FT-IR results.

## 4. Ofloxacin Adsorption at 10 µg L^−1^ Concentration 

The suitability of ZIF-8 and Zn_3_(BTC)_2_ for OFL removal at a few µg per liter concentrations from contaminated water was investigated.

Each MOF (10 mg) was separately added to 20 mL tap water samples spiked with OFL 10 µg L^−1^ (*C*_0_) and stirred overnight. Then, the supernatants were centrifuged and analyzed by HPLC-FD to determine the drug content (*C_t_*). 

The removal efficiency (*r*) was calculated via Equation (10):

(10) $$r=\frac{\left(C_{0}-C_{t}\right)}{C_{0}}\times 100$$

where *C*_0_ is the initial OFL concentration and *C_t_* the OFL concentration after a contact time *t*.

The obtained removal efficiencies were 88 (3) and 72 (3) % for ZIF-8 and Zn_3_(BTC)_2_, respectively. The results underline good antibiotic recoveries with such a small amount of adsorbent and in the presence of matrix constituents. The reusability disadvantage is then negligible, considering the reported advantages.

## 5. Conclusions

In this work, the adsorption properties of two different zinc-based metal-organic frameworks were explored to remove one of the most used fluoroquinolone antibiotic, Ofloxacin (OFL), from polluted water. The experimental results, presented and discussed herein, showed that both ZIF-8 and Zn_3_(BTC)_2_ have a good affinity towards OFL; nevertheless, different mechanisms were involved. Langmuir and sigmoidal models were found to describe better the isotherms adsorption, while pseudo-second-order and sigmoidal models are more suitable to describe the overall kinetic process, respectively. The equilibrium was reached in less than ten minutes for both materials.

Based on XRPD, FT-IR, and SEM analyses, a full investigation allowed us to find the reasons limiting the MOFs re-use. After adsorption in tap water, enhanced degradation of the ZIF 8 structure occurred, while Zn_3_(BTC)_2_ degraded completely. This disadvantage is overshadowed by the satisfactory results obtained in terms of OFL removal efficiency, as high as 88 and 72% for ZIF-8 and Zn_3_(BTC)_2_, respectively, under relevant real conditions, and by the small adsorbent amount used. 

Summing up, the obtained results make these materials excellent candidates for removing FQs from contaminated waters.

## Figures and Tables

**Table 1:** Isotherm parameters were obtained by fitting the experimental data for OFL adsorption onto ZIF-8 and Zn_3_(BTC)_2_.

Adsorption Model	Isotherm Parameters	ZIF-8	Zn_3_(BTC)_2_
	*q_m exp_* (mg g^−^^1^)	88 ± 3	25 ± 3
Freundlich	*K_F_* (mg^(1−*n*)^ L^*n*^ g^−^^1^)	23 ± 6	0.002 ± 0.002
	1/*n* (g min^−^^1^ mg^−^^1^)	0.26 ± 0.06	0.8 ± 0.2
	R^2^	0.879	0.881
Langmuir	*K_L_* (L mg^−^^1^)	0.06 ± 0.03	0.002 ± 0.002
	*q_m_* (mg g^−^^1^)	95 ± 10	66 ± 45
	R^2^	0.902	0.903
BET	*K_s_* (L mg^−^^1^)	0.06 ± 0.03	0.002 ± 0.003
	*K_L_* (L mg^−^^1^)	7.2 × 10^−22^ ± 0	2.1 × 10^−19^ ± 0
	*q_m_* (mg g^−^^1^)	95 ± 11	65 ± 55
	R^2^	0.869	0.855
Sigmoidal	*A*	-	0.034 ± 0.003
	*F*	-	104 ± 4
	*q_m_* (mg g^−^^1^)	-	25.3 ± 0.8
	R^2^	-	0.994

OFL: Ofloxacin; BET: Brunauer-Emmett-Teller ZIF-8: zeolitic imidazolate framework-8; Zn_3_(BTC)_2_: Zn(II) and benzene-1,3,5-tri-carboxylate; *q_m_*: monolayer saturation capacity; *K_F_*: empirical constant indicative of adsorption capacity; *K_L_*: Langmuir constant; *K_s_*: equilibrium constant for the first layer; *A*: sigmoidal efficiency adsorption constant; *F*: sigmoidal flex.

**Table 2:** Kinetic parameters were obtained by fitting the experimental data for OFL adsorption on ZIF-8 and Zn_3_(BTC)_2_.

Kinetic Model	Kinetic Parameters	ZIF-8	Zn_3_(BTC)_2_
	*q_e exp_* (mg g^−1^)	37.6 ± 2	4.9 ± 2
Pseudo-first order	*k*_1_ (min^−1^)	1.2 ± 0.2	0.13 ± 0.05
	R^2^	0.992	0.907
	*q_e_* (mg g^−1^)	37.9 ± 0.4	5.8 ± 0.7
Pseudo-second order	*k*_2_ (g mg^−1^ min^−1^)	0.12 ± 0.04	0.01 ± 0.01
	R^2^	0.993	0.896
	*q_e_* (mg g^−1^)	38.4 ± 0.5	8 ± 2
Sigmoidal	*A*	-	0.9 ± 0.2
	*F*	-	5.3 ± 0.2
	R^2^	-	0.997
	*q_e_* (mg g^−1^)	-	5.31 ± 0.08

*k*_1_: pseudo-first order rate constant; *k*_2_: pseudo-second-order rate constant; *q_e_*: amount of the adsorbed molecule.

## Data Availability

The data presented in this study are available on request from the corresponding author.

## Appendix A

**Table A1:** Physico-chemical characterization of tap water sample

Parameters/Ions		Tap Water
pH		7.7
Conductivity at 20 °C	µS cm^−1^	271
TOC	mg L^−1^	4.2
Cl^−^	mg L^−1^	5.0
NO_3_^−^	mg L^−1^	0.6
SO_4_^2−^	mg L^−1^	5.0
HCO_3_^-^	mg L^−1^	182
Ca^2+^	mg L^−1^	35
Mg^2+^	mg L^−1^	10
Na^+^	mg L^−1^	12
